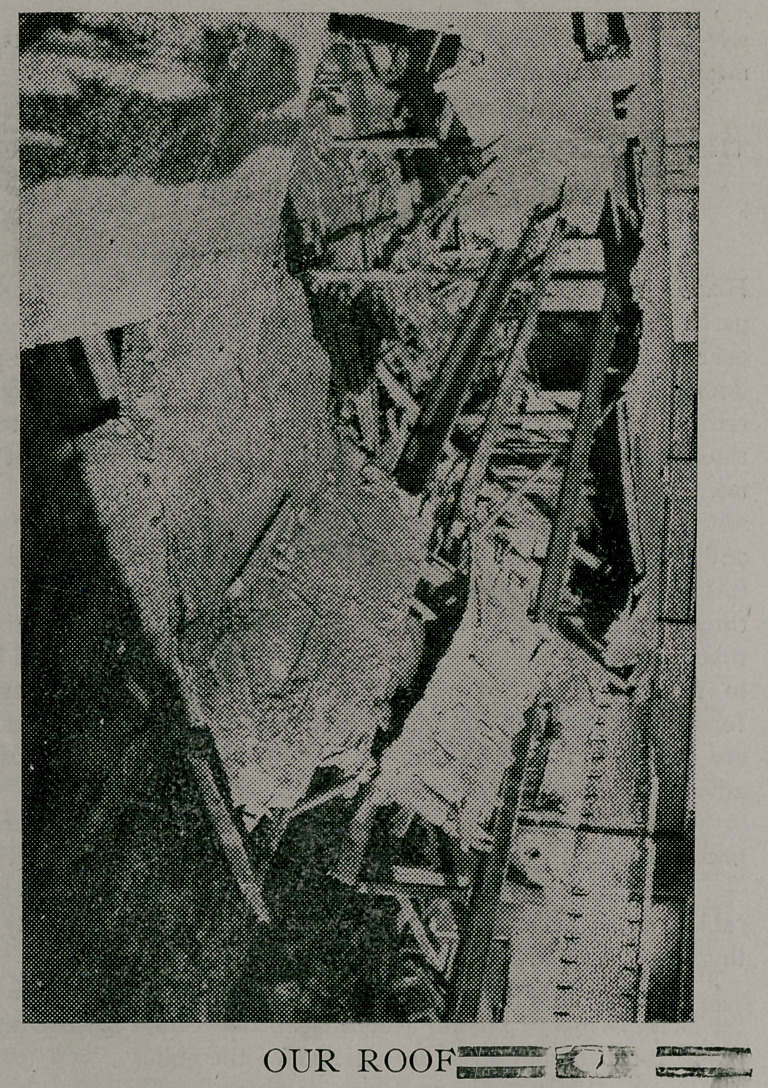# Editorials

**Published:** 1909-01

**Authors:** 


### EDITORIALS

The Business Office of the JOURNAL-RECORD is 1 1-2 to 5 1-2 South Broad Street.
The Editorial Office is 1014-15 Century Building.
Address all Business Communications to *Journal-Record of Medicine*, 1 1-2 to 5 1-2 South Broad Street.
Make remittances payable to *THE JOURNAL-RECORD OF MEDICINE.*
On matters pertaining to the Editorial and Original Communications, address Edgar G. Ballenger, M. D., Atlanta, Ga.
Reprints of Original Articles will be furnished at cost price. Requests for the same should always be made in THE MANUSCRIPT.
We will present, postpaid, on request, to each contributor of an original article, twenty (20) marked copies of THE JOURNAL-RECORD OF MEDICINE containing such article.

### HYSTERECTOMY FOR SPECIFIC INFECTION.

The selection of the proper surgical precedure in the treatment of pelvic disease in the female is a matter of no small difficulty in many cases. Particularly is this true when the disease is of gonorrheal origin. Acute gonorrhea of the female pelvic viscera is not a surgical condition, but with the passing of acute symptoms, and after the most careful and prolonged application of local medication, we will have left a large per cent. of cases in which surgery is absolutely essential. In most of these cases the symptoms that are most urgent are usually attributed, and jutly so, to the involvement of the fallopian tubes and of the ovaries, but we must bear in mind that once gonorrheal infection has spread beyond the internal os of the uterus, that organ suffers as much from its ravages as do the appendages, and is just as certain to be left crippled.

Without taking up the question of conservative work upon the appendages beyond noticing that in such work we take a grave risk of failure of symptomatic cure for a very slight chance of the occurrence of pregnancy, and a still slighter one of successful child-bearing, we will take up briefly the consideration of a condition in which the surgical indications are apparently clear enough, though they are persistently ignored. The performance of double salpingo-oophorectomy without removing the uterus might be justified when all ovarian tissue has been destroyed without disease or displacement of the uterus. However, when the tubes and ovaries have been so damaged by gonorrhea as to necessitate their removal, nothing short of hysterectomy will meet all the surgical indications present.

The uterus is useless as a genital organ without the ovaries. Once the seat of a serious gonorrheal process it is ever afterwards practically useless under all circumstances, and with the ovaries out it is worse than useless, as it is a source of trouble and danger to the patient, and also to others through the infectious discharge coming from it more or less continuously. It is not only impossible to restore the uterus to anything like a normal condition after severe specific infection, but it is also practically impossible to destroy the infective process in the uterine cavity, and to render the uterine flow inocuous.

The necessity for removing this useless and dangerous organ along with the tubes and ovaries will appeal to us at once, unless its removal adds some considerable risk or the organ serves some purpose besides the genital function, which is lost after removal of the appendages, if not already destroyed by the disease. The danger of hysterectomy in practiced hands is very slightly, if at all, in excess of the risk of double salpingo-oophorectomy. Supra-vaginal amputation of the uterus requires little more time than is taken in doing thorough, neat work on the appendages; it leaves a much more satisfactory operative field, as is evidenced by the absence of raw surfaces. It is seldom after removal of the uterus that nearly, if not quite all denuded surfaces cannot be covered with sound peritoneum, and the oeprative field left in the best possible condition to prevent post-operative adhesions with attending discomfort and risk of intestinal obstruction.

Pan-hysterectomy offers advantages over supra-vaginal amputation in that the diseased cervix is gotten rid of. It is a more difficult and somewhat more dangerous operation, and should not be undertaken in this class of cases unless the patient's general condition is good, and the anatomy of the belly wall and of the pelvis lends itself to the procedure. In cases not suitable for pan-hysterectomy almost as good results can be gotten by coring out the cervix, removing the mucus membrane and possibly some of the cervical tissue. This gets rid of that portion of the cervix most apt to give rise to subsequent annoyance.

An objection at times raised to hysterectomy is that the uterus serves an important function as a support to the rectum and bladder, but this is hardly a valid objection, since the weight of the uterus tends to favor its own displacement downward with consequent displacement of both these viscera. Any support it gives to the bladder from behind or the rectum from in front is readily substituted by proper technique in disposing of the remains of the broad ligaments. Without going into further details of this technique it may be explained that the stumps of the round ligaments and of the infundibulo-pelvic ligaments on either side should be drawn down and sutured to the stump of the cervix when it is left in, and to the vaginal wound when the cervix is removed. In this way the vagina is drawn up and supported in position while a diaphragm is constructed across the pelvic that gives as satisfactory support to the bladder and rectum as could be furnished by a normal uterus, much less by a diseased organ already displaced or with a tendency to become so.

This operation which removes only useless organs and which at the same time removes all tissue that is hopelessly diseased and incapable of restoration to anything like normal condition, which leaves a clean operative field without raw surfaces, and which leaves the supporting anatomy essentially intact, appears almost ideal for meeting the operative indications in these cases.. When we add to this the fact that it carries little more risk, if any, than does removal of the appendages alone, we are lead at once to the conclusion that it should be done in every instance in which both appendages need to be removed as the result of gonorrheal infection.

The operator without experience in doing hysterectomy may hesitate through awe for which he considers a much more formidable operative procedure, but practice rapidly produces confidence, and it is soon found that in hysterectomy for inflammatory disease the most difficult part of the work has been accomplished when the appendages have been freed from their pathological attachments. This of course must be thoroughly done in every case no matter which operation is to follow. Inexperience on the part of the operator might well be considered a valid objection to hysterectomy in these cases, but I know of no other and this is one that can be urged on the patient in exceptional instances only.

In specific infection of the uterus and its appendages surgical intervention is rarely indicated during the acute stage. After the subsidence of acute symptoms every effort should be made by prolonged local treatment and careful hygene to restore the organs as nearly as possible to their normal state, and so to avoid operative treatment. When operation proves inevitable the question of partial, so-called conservative, operation upon the appendages arises, a question of great importance which cannot be considered here. When judgment declares in favor of complete removal of both appendages then it is the part of wisdom to remove the uterus as well.

---

### THE SURGICAL PROBLEM.

It is no unusual thing to hear within our own fraternity statements concerning the numerous unnecessary operations which surgeons are said to be doing. "It is a sham" and "it is a crime" we hear it said that this or that was done in the surgical treatment of some case. Rather more charitable in any event where results were the reverse of expectations to have said "an example of error in judgment," "a mistaken diagnosis," rather than it was the result of "a mania for doing operations." or that "mercenary motives were dominant in the decision." While surgeons are human beings with characteristic frailities of their race, they are as a class humanitarians, full of charity and good works, sacrificing themselves for the general good. The same amount of sense, time, and money backed up by the untiring energy and zeal necessary for the development of a good surgeon or a good general practitioner would bring in far greater financial returns in any sphere of the business world, so that on the face of it is born testimony that the medical man is not a good business man, for if he were he would not be a doctor even though he had his license. Many men start out as physicians whose business acumen takes on early development and then they enter other ovenues for the pursuit of wealth. A majority though small, follow the path of an honorable profession, preferring to do good in this special way, to serve the sick and weak, to live a shorter time, but crowding it with service and self sacrifice. That among the rank and file, there are sometimes found those mad for gain of gold as well as fame, that there are even department stores of surgery here and there, is not to be denied, for perfection has not yet become universal in the human race in general nor even among surgeons in particular.

In condemning surgery through the extravagances and extremes of some of the practitioners thereof, one should not be forgetful of the possible harm such a policy might inaugurate if persisted in as seems to be a tendency at present among surgeons themselves. No one individual should feel that he posses-ses the sum total of human discretion. It follows that repeated articles concerning "surgery gone mad" and the "furor operativus" if influencing surgeons alone might be productive of good, yet when added to a long and strong prejudice against surgery among general practitioners, many internists, and more electrotherapeutists and other utists, will be rather far reaching where the influence is finally spent, that is on the general public and their sick.

The surgical situation is really not the mad rush of ruthless removal of diseased organs, nor the ablation of those of doubtful pathology for be it said truthfully that in occasional instances when such is done it is rare that such lasting harm results as compared with the frequent loss of life which follows the refusal of operations from horror of surgery in both patient and general practitioner. Especially is this true of acute inflammatory conditions which often recover under medical treatment only to return in increased severity at some future time, when immediate surgical intervention as soon as the diagnosis is established means a certain cure with a minimum danger, a danger not to be compared with the risks of medical treatment. The real mortality of all acute surgical conditions such as appendicitis, gall bladder inflammations, perforations from ulcers, extra-uterine pregnancies, strangulated hernias, twisted tumor pedicles, intestinal obstructions, etc., arise from delay consequent to imperfect diagnosis, attempts at medicinal measures, and entertaining a hope that the case in hand is of a mild type and that it will be one of those to recover without an operation.

The question every doctor should put to himself is under what treatment do we obtain the higher per cent. of recoveries, in the case in hand, and then let the patient have the benefit of the most hopeful course. It is not a question of whether surgery has gone mad, it is not a question of whether it will benefit or do harm to the attending doctor's reputation, it is a question of human life and health to which no commercial argument should every apply in a decision as to treatment. How many countless thousands have gone to their death because of delayed or no surgery in small tumors of the breast, in neglected lacerations of the cervix, because of medically treated appendicitis, or obstruction, or gall stones, no human statician will ever compute. When the cry is raised against the indiscriminate removal of healthy organs especially the procreative organs of the female and because of past error and present excessive zeal among a few, there is outspoken antagonism to the surgery today is as reasonable as an excuse for crying out against religion because of the error of a few preachers in keeping others from embracing a belief and living up to its helpful tenants, entirely overlooking the fact that it saves many souls, and affords a support at times when every other influence fails.

To the most of doctors who are in practice for the sake of the immeasurable good that lies within them to do for the sick of the human race, there is and can be no other duty but to the patient; fees, reputation, personal comfort and life itself is placed as a stake to overcome disease. Conservatism is a necessity for the continuance of medical and surgical progress, the stability of the profession as a scientific body is dependent thereon. Early diagnosis and immediate operation in those diseases where surgery offers a better statistical cure is conservatism of the best type and makes for progress and helps the education of the public in the knowledge of the usefulness of surgery and robs them of the fear of the knife. This fear is based on the results of surgery deferred till it becomes a last resort and where practiced as such is rarely satisfactory, for it leads to repeated operations and at best a partial cure in most instances. Take the same class of patients and allow them the privilege of early surgery and one operation of comparatively small danger cures permanently.

There is but one problem in the surgical situation, that being the early recognition of surgical diseases and immediate treatment by operation. All else as to extent of operation, removal of organs, of diseased tissues and whether this or that process will recover if left within the body is a matter of personal judgment, of the discrominative power of the individual surgeon based on past experience. The choice between drainage and ablation, between resection and removal, also comes under the surgeon's judgment as to whether this or that tissue has within itself sufficient vitality to further functionate, if not then its removal is conservative.

---

### THE FOURTH ANNUAL REPORT OF THE GEORGIA STATE BOARD OF HEALTH.

---

The Fourth Annual Report of the Georgia State Board of Health has been issued and makes an excellent showing as regards the scientific work being accomplished in its laboratories, particularly the free examination of specimens for disease-producing germs. The report shows that this work is rapdly increasing, and undoubtedly the accuracy and additional interest thus produced will have a beneficial effect on the health of the state by fostering a scientific spirit among her physicians. The untiring efforts of the members of the State Board of Health and especially of Dr. Roy Harris, the secretary, have developed this body into one which wields a wide and wholesome influence throughout the state. They have continued the manufacture of tuberculin which has been extensively and satisfactorily used both in the diagnosis and treatment of tuberculosis. The various forms of tuberculin and their methods of preparation and use are described and physicians are urged to avail themselves of the advantages of this form of medication, in suitable cases.

The Board is now furnishing also free treatment for the prevention of hydrophopia by the method of Pasteur.

When an individual has been bitten by a dog thought to be rabid it is suggested that the wound be cauterized immediately and thoroughly, preferably with concentrated nitric acid.

Diphtheria autitoxin is now prepared in the laboratories of the State Board of Health according to the Gibson method and is ready for distrubution to the citizens of the state.

---

At the annual meeting of the Southern Surgical and Gynecological Association, held in St. Louis, December 15-17, the following officers were elected: Dr. Stuart McGuire, Richmond, Va., president; Drs. John Young Brown, St. Louis, and Robert S. Cathcart, Charleston, S. C., vice-presidents; and Dr. William S. Goldsmith, Atlanta, Ga., treasurer. Hot Springs, Va., was selected as the meeting place for 1909.

OUR ROOF [illegible]

---

### JANUARY ISSUE DELAYED BY FIRE

---

The above picture shows more accurately than words can express the reason for the delay of the present issue of the Journal-Record of Medicine. We ask therefore the indulgence of our patrons for a few months until we are again in thorough working order.

## Figures and Tables

**Figure f1:**